# Advances in seed hypoxia research

**DOI:** 10.1093/plphys/kiae556

**Published:** 2024-10-29

**Authors:** Hardy Rolletschek, Ljudmilla Borisjuk, Eva María Gómez-Álvarez, Chiara Pucciariello

**Affiliations:** Leibniz Institute of Plant Genetics and Crop Plant Research (IPK) Gatersleben, 06466 Seeland, Germany; Leibniz Institute of Plant Genetics and Crop Plant Research (IPK) Gatersleben, 06466 Seeland, Germany; PlantLab, Institute of Plant Sciences, Scuola Superiore Sant'Anna, 56010 Pisa, Italy; nanoPlant Center @NEST, Institute of Plant Sciences, Scuola Superiore Sant'Anna, 56127 Pisa, Italy; PlantLab, Institute of Plant Sciences, Scuola Superiore Sant'Anna, 56010 Pisa, Italy; nanoPlant Center @NEST, Institute of Plant Sciences, Scuola Superiore Sant'Anna, 56127 Pisa, Italy

## Abstract

Seeds represent essential stages of the plant life cycle: embryogenesis, the intermittent quiescence phase, and germination. Each stage has its own physiological requirements, genetic program, and environmental challenges. Consequently, the effects of developmental and environmental hypoxia can vary from detrimental to beneficial. Past and recent evidence shows how low-oxygen signaling and metabolic adaptations to hypoxia affect seed development and germination. Here, we review the recent literature on seed biology in relation to hypoxia research and present our perspective on key challenges and opportunities for future investigations.

## Introduction

A seed is a mature ovule containing an embryo (a miniature and underdeveloped plant) and food reserves, all of which are enclosed by protective layers (seed coat and fruit tissue). During early seed development, mitotic activity is very high and is accompanied by high mitochondrial respiration (Borisjuk et al. 2003; Rolletschek et al. 2003). When the seed grows, it accumulates storage reserves and becomes a densely packed organ, sometimes covered with specific sealing layers that further restrict oxygen diffusion (Herzog et al. 2023). It is not surprising, therefore, that oxygen deprivation can occur. Toward the end of its development, the seed desiccates and may become quiescent, thus reducing its oxygen demand. However, when the seed awakens after imbibition, that is, germinates, the mitochondria are reactivated within minutes (Nietzel et al. 2020), leading to a large increase in the respiratory oxygen demand, which again creates the “likelihood” of hypoxia. Hypoxia can also be induced by the environment, for example, flooding events. Such “environmental hypoxia” can affect germination in species-specific ways.

These simple descriptions reflect what decades of research in the field of seed biology have revealed. Why is this research important? Seeds are the basis of human and animal life on Earth. The food we eat, the clothes we wear, and many of the products we use in our daily lives come from seeds. Hypoxia, which is traditionally considered a stressor, can interfere with the seed's mission, limiting the synthesis of storage products, germination success, and ultimately crop yield.

We need to find answers to some crucial questions: Is hypoxia truly a stressor or rather the norm during seed development and germination? What specific structures determine the access of gaseous oxygen into the seed? What are the regulatory genes and biochemical adaptations to hypoxia in developing and germinating seeds? These and other questions are addressed in the following chapters. Answers to these questions are not only of academic interest but also allow very specific strategies to be derived: what happens in the event of externally increased hypoxic stress, how can the plant respond, and how can this be translated by breeding/biotechnology into stress-tolerant crops with stable high yields? A close look at seeds is therefore worthwhile in many respects.

## Oxygen deficiency is the normal condition in developing seeds

**Figure 1.**
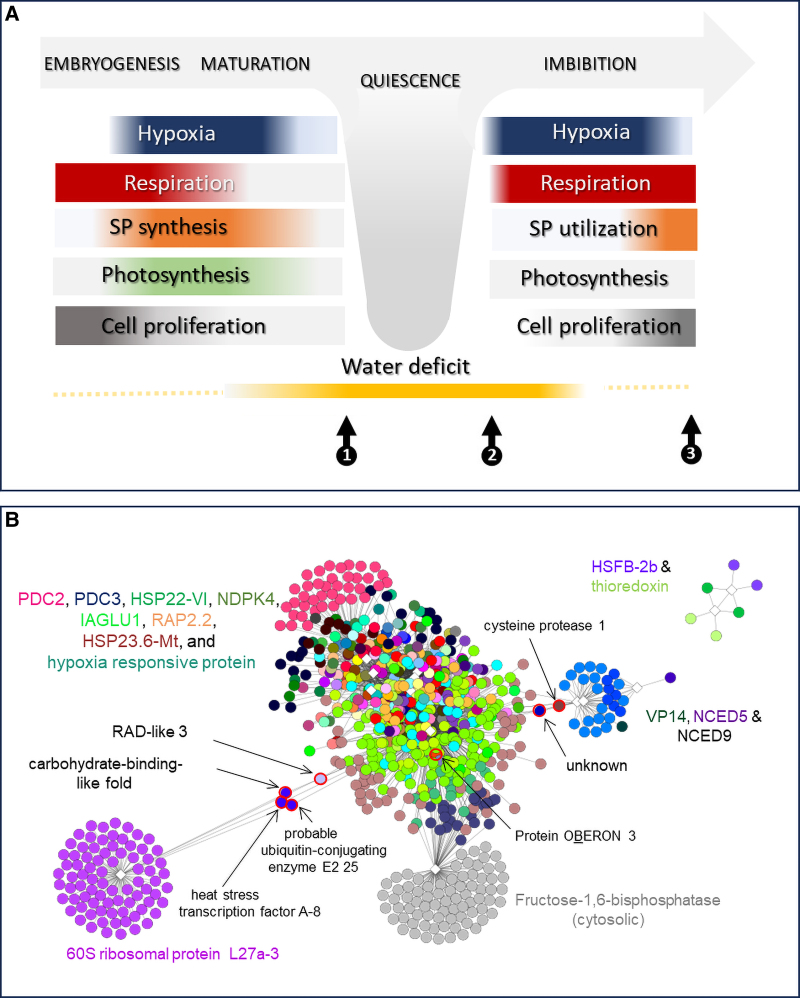
Developmental transitions of seeds in relation to endogenous hypoxia and physiological activities. A) Early development is characterized by high mitotic and metabolic activity, which induces endogenous hypoxia. During the subsequent seed filling phase, storage products (SP) are synthesized and respiratory activity is maintained at a high level, which also maintains the hypoxic state of the seed tissues. This changes as seeds begin to desiccate during late maturation, with a rapid decline in both water content and metabolic activity, while oxygen levels equilibrate with the environment (atmospheric normoxia). The seeds of most species undergo a period of quiescence (1), during which metabolic activity approaches zero. As a result, the oxygen level of the seed is fully equilibrated with atmospheric (normoxic) levels. Upon imbibition (2), water content and respiratory activity increase sharply, accompanied by a high oxygen demand and the re-establishment of endogenous hypoxia. The emergence of the radicle through the seed coat marks the end of germination (3). As the sealing layers of the seed are broken, oxygen can freely diffuse into embryonic tissues, accompanied by a marked increase in tissue oxygenation. Hypoxic niches may remain and are important for plant growth and differentiation. B) Gene coexpression networks found in developing maize kernels. Networks and their relationships were defined from the 1,222 genes that responded to oxygen perturbation using 15 bait genes selected from this group based on their known roles in other systems. Dots with the same color represent genes coexpressed with a given bait. Five coexpression modules emerged: 1 large cluster included respiration, stress responses, and biogenesis of cellular components. Three of the other modules were distinct from but related to this large cluster. One was defined by the ribosomal L27a-3 bait and included other nucleosome- and ribosome-related mRNAs. The second module included the 2 bait genes, viviparous14 and NCED5, as well as NCED9, which together indicated a level of coordinated expression between the large respiratory cluster and genes encoding ABA biosynthesis/sensing. The third module defined a separate source of respiratory input, delineated by a gene for the glycolytic enzyme cytosolic fructose-1,6-biphosphatase(F-1,6 BPase) and transcripts for mitochondrial membrane proteins. For further details, see (Langer et al. 2023).

Hypoxia refers to oxygen concentrations substantially below normoxic levels (20.9 vol%). The core findings regarding seed hypoxia have been acquired using microsensors that provide high-resolution oxygen maps. Minimum oxygen levels are usually reached during the early to mid-developmental stages in the seed’s center of all major crops (cereals such as maize, barley, and wheat; various legume species and oilseeds) (Borisjuk and Rolletschek 2009). Oxygen concentrations typically decrease to <10 *µ*mol/L (7.3 hPa or 0.7% vol/vol), which corresponds to approximately 97% deficiency compared with normoxia. The level of hypoxia varies substantially with developmental stage and roughly correlates with overall metabolic activity (Fig. 1A). Mitochondrial respiratory demand is regarded as the major oxygen sink in seeds. However, respiration alone does not always explain the extent and the spatial pattern of hypoxia. For example, the intensely respiring maize embryo can avoid oxygen deficiency, while the endosperm (which is less active in respiration at the respective stage) becomes severely hypoxic (Borisjuk and Rolletschek 2009). Thus, additional factors (such as diffusion barriers) contribute to the local onset of hypoxia.

The recent meta-analysis by Herzog et al. (2023) identified “submergence,” “light,” and “tissue type” as significant influences on plant tissue oxygen status. This is also partially true for developing seeds, especially for light and tissue type/development stage (whereas submergence is usually not relevant). Comparing seed hypoxia with that reported for other plant tissues (Herzog et al. 2023), it is clear that seed tissue oxygen levels are at the lower end of the scale. Thus, severe oxygen deficiency is the normal condition in developing seeds. Despite such generalizations, the occurrence of steep oxygen concentration gradients should not be overlooked. Tissue oxygen status inside the seed can range from undetectable levels (<0.1 *µ*mol/L or true anoxia) to more than 2.5 times atmospheric equilibrium, as demonstrated for barley grains (Rolletschek et al. 2004).

## What specific structures determine and limit the access of gaseous oxygen to the developing seed?

Much progress recently has been made in analyzing the oxygen diffusion capacity of seeds by the combined use of X-ray micro-computed tomography (µ-CT), magnetic resonance imaging, oxygen profiling, and reaction-diffusion modeling. Seeds appear to have specific structural elements that either restrict or accelerate gas diffusion and thus control the oxygen supply to the interior (video 1). For example, a recent study on developing maize kernels demonstrated that the chalazal pericarp harbors a highly porous layer, which is important for oxygen balance in kernels (Langer et al. 2023). Similar void networks with interconnected pores have been found in oilseeds (Cloetens et al. 2006; Verboven et al. 2013) and several seed-bearing fruits (Ho et al. 2011; Xiao et al. 2018, 2024). The functional significance of voids is based on the fact that oxygen diffusion in air is approximately 10,000 times faster than that in water.

Void networks not only enable high rates of gas exchange but also determine the preferred diffusion path inside the seed: in the case of maize, most of the oxygen enters the kernel via its placental chalazal region (Langer et al. 2023). This study also revealed that oxygen diffusion is not hampered by the outer cuticle/pericarp (as expected), but the endosperm itself constitutes a major limitation to diffusion. Its cells are densely packed with starch, it has no intercellular voids, and the progressive crushing of nucellar cells (and inner integument) likely produces lipidous barrier material at the endosperm surface, which additionally hampers gas exchange. In seeds of *Brassica napus*, the void network is restricted to the embryo radicle, enabling preferential diffusion along this axis (Verboven et al. 2013), while the cotyledons are rather dense (low diffusion rate).

Does the void space represent a significant storage space for oxygen? Modeling results argue against this hypothesis. In the case of *B. napus*, the amount of oxygen stored in the voids is sufficient for merely 1 minute of normal seed respiration. The interpretation of voids in *Arabidopsis* (Cloetens et al. 2006) has remained difficult because only mature (dry) seeds have been studied, leaving open the question of whether these networks are filled with gas or water during seed development.

Structural and gas diffusion modeling of fruits (Xiao et al. 2018, 2024) has revealed that the tissue regions inside the fruit where seeds are located have the lowest oxygen concentrations within the entire fruit. The implications are that (1) seeds are relevant oxygen sinks inside the fruit; and (2) the oxygen concentration gradient (needed to drive diffusion) is quite flat, which severely restricts the oxygen availability to the seeds. This scenario thus resembles a waterlogging/submergence scenario. The seed-fruit relationship is an exciting and largely unexplored area of hypoxia research with the potential to improve crop quality (Simkin et al. 2020; Lawson and Milliken 2023).

## What do we know about low-oxygen sensing/signaling in developing seeds?

Early-stage seeds comprise very active meristematic tissues, with some similarity to the mitotically active cell niches in shoot and root meristems, which all tend to be hosted at low oxygen tension. A variety of oxygen-, redox-, and energy-dependent changes (Lee and Bailey-Serres 2019; Wagner et al. 2019; Meng et al. 2020; Sasidharan et al. 2021) contributes to the low-oxygen response of these tissues. Specific transcription factors, such as group VII ethylene response factors (Loreti et al. 2016), VERNALISATION 2 (VRN2), and LITTLE ZIPPER 2 (ZPR2), are involved in plant development (reviewed by Weits et al. 2021), as is ANAC13 (Eysholdt-Derzsó et al. 2023). None of these transcription factors have been functionally characterized in the context of seed development. However, it seems reasonable to assume that the basic principles of low-oxygen sensing and signaling also apply to seeds. Key genes such as those encoding plant cysteine oxidases and Class VII-Ethylene Response Factors (ERFs) are expressed during seed development, and some of these genes respond to experimental modulation of the oxygen supply (Langer et al. 2023). The same applies to some of their target genes with appropriate promoter motifs (Gasch et al. 2016). The remarkable convergence of functional features for low-oxygen sensing/signaling among higher plants and animals (Hammarlund et al. 2020) is further supportive of the above assumption.

Much research has been carried out on nitric oxide (NO) in seeds, its interplay with low-oxygen sensing, and its manipulation via modulated phytoglobin (PGB) expression in seeds (Thiel et al. 2011; Hebelstrup et al. 2014; Manrique-Gil et al. 2021). The metabolic effects of PGB overexpression in seeds seem to partially differ from those in roots (Mira et al. 2023)

ADVANCES BOX

- The availability of genetic resources coupled with geographic information on plant accessions has led to the identification of new molecular mechanisms involved in the response of plants to hypoxia. Substantial information is available, such as for Arabidopsis (https://www.1001genomes.org/) and rice (http://gigadb.org/dataset/200001).
- The use of large collections of plant accessions containing landraces and wild relatives favors the study of traits related to hypoxia tolerance from an evolutionary perspective.
- CRISPR/Cas9 techniques support the development of precise transgenic crop lines to study the function of key genes involved in the hypoxia response.
- Metagenomic analysis and functional profiling tools support the study of microbiome composition and dynamics under seed developmental and environmental hypoxia.
- Developments in the field of X-ray CT and magnetic resonance imaging have led to the non-invasive imaging and modelling of seeds.

## Primary effects of hypoxia during seed development

Functions affected by low internal oxygen cover assimilate uptake, central metabolism, and storage activity (Geigenberger 2003; Borisjuk and Rolletschek 2009). Seed photosynthesis is important for sustaining aerobic metabolism under severe hypoxia (Borisjuk et al. 2020; Shackira et al. 2022; Brodersen and Kühl 2023), with implications for seed fitness even in tiny *Arabidopsis* model seeds (Allorent et al. 2015). Hypoxia also seems to affect the rate of seed development as recently revealed for maize kernels (see Box 1).

BOX 1. **How and why is the seed development rate slowed by endogenous hypoxia?**

Recent work on maize kernels indicates that a long-term elevated oxygen supply enhances the rate of development (Langer et al. 2023). This finding was surprising, and let us speculate that, vice versa, hypoxia (associated with a decrease in respiration) slows down regular seed development. Intriguing analogies have been highlighted in studies on animal embryogenesis, where researchers found that the rate of development correlated with mitochondrial activity (Diaz-Cuadros et al. 2023). The cytosolic NAD^+^/NADH redox balance (but not the cellular energy charge) scales with the developmental rate. NAD^+^ is used as an electron acceptor in many metabolic reactions. Its impaired regeneration under hypoxia could be congruent with the proposal that a lower NAD^+^ to NADH ratio slows development. Similar findings were shown in another study using animal cells (Iwata et al. 2023). Delayed cellular maturation was observed with treatments that decreased mitochondrial metabolism (and vice versa). This scenario is synonymous with hypoxia. A major metabolic category downstream of mitochondrial activity is the protein translation rate (Diaz-Cuadros et al. 2023), which is likewise among the most affected processes in hypoxia-treated plant seeds. From this perspective, chronic hypoxia during seed development in plants may serve to decelerate the growth process. Why should the plant do this? We can only speculate that this is because, for example, slowing the developmental rate includes lower mitotic rates, providing more time for DNA synthesis/repair, which may eventually lower the risk of introducing mutations. Many more processes may benefit from oxygen levels below normoxia, and plants may employ unexpected mechanisms to adapt to varying oxygen partial pressures (Abbas et al. 2022). More research is needed to clarify the potentially favorable adjustments in seed growth and fitness due to hypoxia. Inspiration may come from the medical field, which has shown that controlled phases of hypoxia, such as those used in altitude training, can bring about positive adaptations and health benefits (Sghaier-Hammami et al. 2020).

### Mitochondrial pathways

Mitochondrial adjustments to endogenous hypoxia are of prime importance in developing seeds (Fig. 1B) and are evident at both the biochemical and gene expression levels. Oxygen responsiveness involves voltage-dependent anion channel proteins, which control metabolite trafficking between mitochondria and the cytosol, as well as heat shock proteins, which control both the electron transport chain and respiratory activity (Langer et al. 2023).

The balancing of mitochondrial activity has multiple implications: (1) stabilizing the seed’s steady-state oxygen levels and thus preventing anoxia (Rolletschek et al. 2011); (2) contributing to metabolic acclimation; and (3) the acclimation of mitochondrial functions as sensors of stress and signaling hubs. Chronic hypoxia in seeds induces GABA-shunt activity but with an open loop leading to the accumulation of 4-hydroxybutyrate (Shelp et al. 2017; Langer et al. 2023). This is likely connected to the transcriptional activation of genes encoding 4-hydroxybutyrate dehydrogenase (Breitkreuz et al. 2003).

Mitochondrial pathways and energy fluxes impose constraints on major cellular functions (Yang et al. 2021). Thus, adjusting mitochondrial activity to oxygen availability is of highest relevance. The low-oxygen response is generally tightly linked to mitochondrial energy signaling (Schmidt et al. 2018; Wagner et al. 2019) and its mediators SnRK1 (Wurzinger et al. 2018) and TOR (Kunkowska et al. 2023). The involvement and exact role of these mediators in the context of seed hypoxia still needs to be clarified. The plant tricarboxylic acid (TCA) cycle was previously shown to operate in noncyclic flux modes upon seed hypoxia (Rolletschek et al. 2011). In addition to their function in respiration, TCA cycle enzymes exhibit many extra-pathway interactions (Zhang and Li 2017; Zhang and Fernie 2023). Their analysis is expected to provide important insights into how hypoxic seeds partition their resources among different cellular processes.

### Glycolysis and fermentation

Energy metabolism also occurs outside mitochondria, namely via glycolysis and fermentation. These pathways are thus elements of hypoxic acclimation. Genes encoding glycolytic (and TCA cycle) enzymes have been found to be upregulated in the less hypoxic seed periphery, while genes involved in fermentative metabolism were upregulated in the hypoxic seed center (Gayral et al. 2017). Such spatial patterns in seed metabolism are important elements of hypoxic acclimation. Metabolic regulation allows for increased ATP production in the endosperm periphery (and embryo), where energy-intensive protein (and lipid) synthesis preferentially occurs. Although this may be coincidental, there is a clear functional and evolutionary advantage for the sites of processes (lipid and protein storage) that are most restricted by hypoxia (energy depletion) to be located in the peripheral tissues of cereal grains.

In contrast to vegetative tissues, seeds of many species have completed glycolytic pathways in both the cytosol and plastid compartments. It has been proposed that this process enables high metabolic flexibility and high glycolytic flux, especially essential for fatty acid synthesis in oilseeds. Many plant species use the often-overlooked Entner-Doudoroff pathway for glycolysis instead of the commonly considered Embden–Meyerhof–Parnas pathway (Chen et al. 2016). Although the Entner-Doudoroff pathway has a lower ATP yield, it also has lower protein costs and avoids futile cycles compared with the Embden–Meyerhof–Parnas pathway. Determining which of these alternative pathways for sugar degradation are actually used could be relevant in terms of seed hypoxia tolerance and avoidance.

The transcriptional upregulation of genes encoding the fermentation enzymes alcohol dehydrogenase and lactate dehydrogenase in developing seeds is evident under hypoxia treatment (as in other plant organs). However, this does not seem to necessarily translate into substantially elevated pathway fluxes. This indicates the operation of an avoidance mechanism, but the underlying mechanisms (e.g. redox control; Dumont et al. 2018) have not been investigated in seeds. Notably, seeds utilize other means of fermentation metabolism mediated by sorbitol dehydrogenase (Sdh) and alanine aminotransferase (AlaAT). In maize, the single copy gene Sdh1 is essentially endosperm specific, where its capacity to form sorbitol could aid in redox balance and maintenance of energy metabolism, especially under hypoxic conditions in the endosperm. The activity of AlaAT in cereal seeds has been well characterized, improving the carbon and energy economy in grain (Rolletschek et al. 2011; Borisjuk et al. 2020), regulating starch storage (Yang et al. 2015), and controlling dormancy (Sato et al. 2016). Much less is known about the metabolic contribution of acetate fermentation in seeds (Jardine and McDowell 2023). Acetate fermentation could have several advantages for hypoxic oilseeds: (1) acetate biosynthesis helps in the regeneration of NAD^+^ required to maintain a high glycolytic flux; (2) it contributes to a metabolic shift away from the direct entry of pyruvate into the TCA cycle (which is partially blocked under hypoxia); and (3) acetate could be used as an immediate metabolic precursor for fatty acid synthesis. Recent work on cancer cells has demonstrated that acetate functions as an epigenetic metabolite capable of promoting both lipid synthesis and cell survival under hypoxia (Gao et al. 2016).

## Do quiescent seeds require oxygen or is it better for them to avoid oxygen?

Most seed plants have what are known as orthodox seeds. These seeds undergo a severe desiccation phase (as opposed to recalcitrant seeds) (Leprince et al. 2017). Desiccation brings the embryo to a quiescent (dormant) state, where it is not assumed to be actively respiring or demanding oxygen. Without endogenous oxygen consumption, an equilibrium with the external atmosphere is inevitably established over time; that is, dormant seeds are likely to be normoxic when in contact with the atmosphere but rather hypoxic when buried in natural seed banks (soil). Storage of dry, mature seeds in ex situ gene banks sometimes occurs under an artificial, low-oxygen atmosphere, highlighting the beneficial effects of hypoxia during this phase (Groot et al. 2015). Hypoxia improves seed longevity, whereas normoxia causes oxidative damage to storage lipids, membranes, and proteins (Wiebach et al. 2020). It is worth noting that our knowledge of potential (residual) activities of dormant seeds during storage is very limited due to technical limitations. During seed storage, some enzyme-catalyzed pathways are likely to operate, although at a greatly reduced rate. The physical state of the cytoplasm appears to be important (Gerna et al. 2022). This in turn may have significant implications for the use of commonly used accelerated aging protocols to understand the mechanisms of viability loss in seed stored in seed banks. The mechanisms underlying metabolic quiescence and desiccation tolerance in seeds have some similarities to those observed in resurrection plants (Costa et al. 2017). Further investigations are urgently needed as they may hold the key to drought tolerance and crop improvement.

## Waterlogging and flooding lead to seed hypoxia

Seed germination, which encompasses events from imbibition to radicle protrusion, can be severely affected by flooding events. Important exceptions are plants that are genetically adapted to a developmental program that seems to be specific for hypoxic conditions (Table 1). The oxygen level is not a limiting factor when the soil is well drained; however, its reduction can be drastic when the soil pore system is filled with water (Sharma and Kumar 2023). Germination depends on the mobilization of reserves to feed the sink organs; thus, the availability of energy sources is vital in a developmental stage where the photosynthetic machinery is still inactive (Carrera-Castaño et al. 2020). Under hypoxia, most cereal seeds are unable to mobilize reserves from the endosperm because crucial molecular processes cannot occur without O_2_ (Loreti and Perata 2020). In fact, α-amylases finalized to hydrolase starch and responding to oxygen shortage are expressed only in rice (Guglielminetti et al. 1995).

**Table 1.** Genes regulating cereal germination under hypoxia and anoxia

Gene	Gene annotation	Species and function	First reference
*OsAmy3D*	α-Amylase 3 D	Rice, starch use	(Hwang et al. 1999)
*OsMYBS1*	MYB sucrose 1	Rice, starch use	(Lu et al. 2002)
*OsSnRK1A*	Sucrose nonfermenting-1-related protein kinase 1	Rice, starch use	(Lu et al. 2007)
*OsCIPK15*	CBL-interacting protein kinase	Rice, starch use	(Lee et al. 2009)
*HvABI5*	ABA insensitive 5	Barley, dormancy	(Mendiondo et al. 2010)
*OsAdh1*	Alcohol dehydrogenase 1	Rice, coleoptile length	(Takahashi et al. 2011)
*OsTPP7*	Trehalose-6-phosphate phosphatase	Rice, starch use	(Kretzschmar et al. 2015)
*OsCBL4*	CBL 4	Rice, starch use	(Ho et al. 2017)
*OsTIR1*	Transport inhibitor response 1	Rice, coleoptile length	(Guo et al. 2016)
*OsCBL10*	CBL 10	Rice, starch use	(Ye et al. 2018)
*OsAUX1*	Auxin transport 1	Rice, coleoptile length	(Nghi et al. 2021)
*OsGF14h*	14-3-3 protein-coding	DW rice, germination	(Sun et al. 2022)
*OsUGT75A*	UDP-glucosyltransferase	Rice, coleoptile length	(He et al. 2023)
*HvLAC*	Laccase	Barley, dormancy	(Gómez-Álvarez et al. 2023)

CBL, calcineurin B–like.

The ability of plants to germinate under low O_2_ conditions was studied long ago (reviewed by Corbineau 2022). A seed group sensitive to an O_2_ concentration below 2% was identified, which included cabbage, carrot, lettuce, radish, rape, sunflower, and turnip. An intermediate group was composed of species that are sensitive to an O_2_ concentration lower than 1%, which includes chicory, sugar beet, leek, melon, and tomato. The most tolerant group corresponded to species mainly composed of starchy seeds, such as barley, wheat, maize, oats, and sorghum, with rice being the most tolerant.

However, a large variation in tolerance is observed when studying collections of plant accessions composed of different genotypes. One example is the evaluation of the germination tolerance to hypoxia and waterlogging of some soybean genotypes that show wide variability (Nguyen et al. 2021, 2023). One crucial aspect when studying plant adaptation is the fact that oxygen deficiency can be beneficial for the germination of some seeds. In fact, some wetland species, such as some *Nymphaea* spp. (Dalziell et al. 2019) and mudflat species (Phartyal et al. 2020), require low oxygen to break dormancy and promote successful germination. In addition, dormant seeds of not wetland species, such as apple (Côme et al. 1985) and some accessions of *Oldenlandia corymbose* (Corbineau and Côme 1985), display a better germination under anaerobic conditions while increased oxygen level dampens germination.

## Understanding how rice can germinate under water

Anaerobic germination tolerance, which allows homogeneous germination and establishment of the seedling under submergence, is not a very common feature in crops. Most cereals, such as barley, wheat, oats, and rye, cannot germinate under complete anoxia, possibly because they lack the necessary enzymes for starch breakdown without O_2,_ due to the inefficiency of the fermentative metabolism (reviewed by Gómez-Álvarez and Pucciariello 2022). However, rice is one exception since it can elongate coleoptiles, which, under submergence, enables the plant to reach the water surface to allocate oxygen to submerged plant parts. This then re-establishes aerobic respiration, thus meeting the energy requirements of growing leaves and roots and full plant development (reviewed by Pucciariello 2020).

Under submergence, the pathway that converges sugar starvation and hypoxic signals culminates with starch degradation and the mobilization of soluble sugars from the endosperm to sink organs. In fact, rice harbors α-amylase 3, which is expressed under anoxic conditions and is independent of the aerobic GA promotion. In turn, low-O_2_–sensitive cereals such as wheat and barley fail to induce α-amylase 3 under anoxic conditions (reviewed by Adak et al. 2023). De novo GA biosynthesis is probably not active under O_2_ shortage because it requires O_2_ in several steps. Moreover, rice contains 4 α-amylase 3 encoding genes, while barley and wheat harbor only 1 each (Zhang and Li 2017), which may contribute to a reduced efficiency in the absence of oxygen. Following the model proposed by Lee et al. (2009), the hypoxia-dependent Ca^2+^ signal operates through a calcineurin B-like protein (CBL) and (CBL)-interacting protein kinase (CIPK) system to start the signaling cascade. The downstream events of the CIPK15-CBL interaction include the regulation of sucrose-non-fermenting-1-related protein kinase 1A (SnRK1A) and the transcription factor MYBS1 (Lu et al. 2002, 2007). MYBS1 binds to the promoter of α-amylase 3 to promote starch hydrolysis in the endosperm under O_2_ shortage. In this pathway, experiments with rice protoplasts have suggested that CBL4 is a positive regulator of the signaling cascade (Ho et al. 2017), while CBL10 was described as a negative regulator. In fact, natural variation in the CBL10 promoter was associated with lower CBL10 expression under oxygen shortage conditions and higher α-amylase 3 expression (Ye et al. 2018).

A further regulator of the cascade that culminates in starch degradation in rice seed endosperm is the trehalose 6 phosphate phosphatase 7 gene (*TPP7*) (Kretzschmar et al. 2015). *TPP7* encodes an enzyme that converts trehalose 6 phosphate (T6P) into trehalose, thus modifying the local sucrose and T6P balance. This boosts α-amylase expression and improves the source-to-sink movement of soluble sugars, enabling coleoptile elongation and improving seedling establishment. Recently, *OsTPP7* was studied in a large collection of Korean rice accessions, and 3 major haplotypes were identified, of which the one belonging to the *Japonica* accessions exhibited the highest anaerobic germination tolerance (Aung et al. 2023).

In some *japonica* rice varieties, the length of the coleoptile depends on auxin transport through AUX1, which promotes elongation (Nghi et al. 2019, 2021). The escape strategy of elongating the coleoptile to reach the water surface is thus mediated by both sugar translocation and possibly cell expansion, which depend on auxin transport. In fact, auxin receptor transport inhibitor response 1 (TIR1) and auxin signaling F-box2 (AFB2) are enhanced during rice coleoptile elongation due to the degradation of *miR393*. This miRNA, which degrades TIR1 and AFB2 in Arabidopsis (Si-Ammour et al. 2011), is present under aerobic conditions but is degraded under submergence (Guo et al. 2016).

A regulation of anaerobic germination was recently identified in weedy rice, which has long been considered an interesting genetic source due to its high resistance to environmental cues and relevant yield quality (Wu et al. 2022). In this rice, the 14-3-3 protein-coding gene *OsGF14h* increases anaerobic germination tolerance, affecting the abscisic acid (ABA)/GA balance (Sun et al. 2022). Subsequently, a further regulator of ABA and JA was identified that accelerates coleoptile growth, decreasing the phytohormone-free level through glycosylation (He et al. 2023). In fact, the glucosyltransferases encoding gene OsUGT75A, which catalyzes the glycosylation of indole-3-acetic acid, indole-3-butyric acid, and ABA in *Arabidopsis* (Zhang et al. 2016; Chen et al. 2020), operates through the glycosylation of ABA and JA under submergence, the free availability of which is then limited, promoting coleoptile elongation.

Finally, the involvement of the PGB/NO cycle contributes to energy availability during deepwater rice germination (Kumari et al. 2022). The supply of nitrite to the submerged water, which supports the activation of the PGB/NO cycle, enhances the capacity of rice to germinate under these conditions. In fact, this cycle leads to the production of small amounts of ATP through the reduction of nitrite to nitric oxide at mitochondria when oxygen is low (Gupta et al. 2022).

## Hypoxia can induce secondary dormancy in seeds

Oxygen deficiency during germination can be detrimental to barley seeds (Arduini et al. 2016). This is probably due to the lack of α-amylase, which works independently of GA availability but is specifically expressed when O_2_ is unavailable, as in rice. The inability to use starch reserves de facto precludes germination until GA-dependent α-amylases can function.

**Figure 2.**
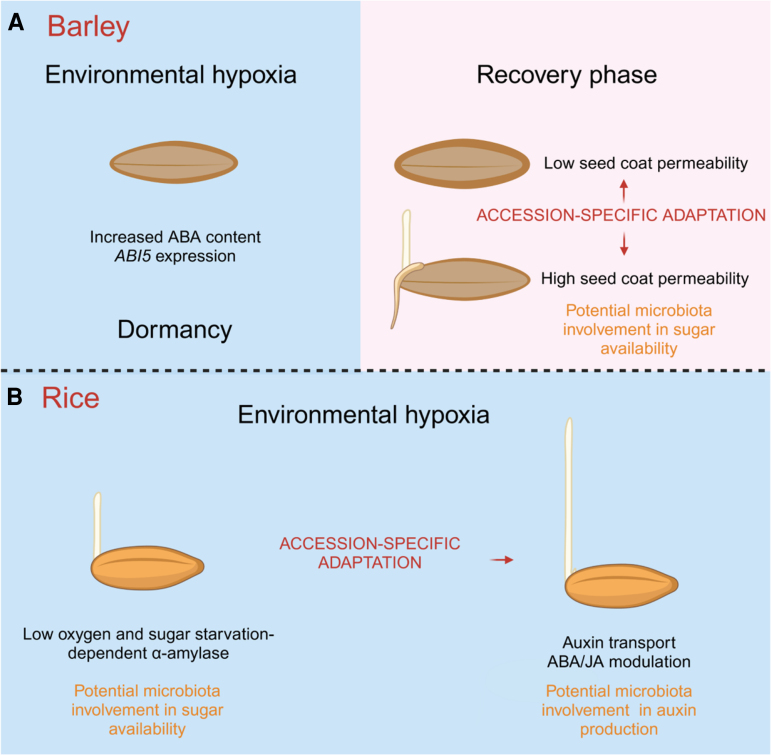
Environmental hypoxia during cereal seed germination. **A)** During cereal germination, if exogenous hypoxia is set, only rice will be able to promptly germinate, while barley and possibly other species, may enter secondary dormancy via an increase in ABA content and ABI5 expression. Under hypoxia, GA-dependent α-amylases are likely not promoted precluding starch degradation. However, some barley accessions with highly permeable seed coats can germinate post submergence. They also possess an enriched bacterial community that can potentially contribute to boost germination through sugar use. **B)** When hypoxia occurs, rice can promptly germinate due to the presence of α-amylase, which is independent of GA and is instead triggered by sugar starvation and low oxygen. Microbiota can potentially contribute to starch degradation. Some rice accessions display an escape strategy with the appearance of a long coleoptile where auxins, ABA and JA modulation are involved and a bacterial community that has the potential to support elongation. Created in BioRender.com.

Under submergence, recent evidence suggests that the reduced capacity to degrade starch reserves is likely coupled, in some barley genotypes, with the possible activation of a secondary dormancy state (Fig. 2). Seeds can be subjected to different types of dormancy (Considine and Considine 2016). Secondary dormancy is the result of unfavorable environmental conditions that include hypoxia, such as flooding events. However, hypoxia can also be an indirect result of physical barriers since seed covering structures such as the glumellae, pericarp, and seed coat as well as thick and lignified structures can physically limit O_2_ availability for the embryo (Pucciariello and Perata 2024). Polyphenol oxidase possibly available on the seed surface consumes O_2_ (Taranto et al. 2017), further reducing diffusion.

The activation of hypoxia-induced secondary dormancy in barley grains has been correlated with the modulation of the hormonal balance of ABA/GA. During barley seed imbibition under hypoxic conditions, the decrease in embryonic ABA is slower than that in air (Mendiondo et al. 2010; Hoang et al. 2013).

The incubation of primary dormant barley embryo grains with exogenous ABA showed that hypoxia increased the sensitivity to ABA by 2-fold (Benech-Arnold et al. 2006). Dehulled barley grains under hypoxia behave similarly to dormant hulled grains since they exhibit dormancy and higher ABA content, suggesting that hypoxia is involved in dormancy in hulled grains (Mendiondo et al. 2010; Cueff et al. 2022).

A recent study showed that although barley is unable to germinate under complete submergence, there is variability in germination in the recovery phase after submergence (Fagerstedt 2023; Gómez-Álvarez et al. 2023). Barley accessions able to promptly germinate post submergence have more permeable and less lignified seed coat, which enables O_2_ diffusion from water to the embryo during submergence. The avoidance of hypoxia-induced secondary dormancy in the germinating barley accessions was correlated with reduced ABA content and signaling.

The PGB/NO cycle is important for regulating the response of barley to short periods of submergence stress during germination (Gupta et al. 2022). Pgb1 is normally induced in the early stages of barley germination before radicle protrusion, when the seed is hypoxic due to the rapid consumption of O_2_ (Zafari et al. 2020). Pgb1 is expressed under hypoxia and is involved in scavenging NO (Nie et al. 2022). Under aerobic conditions, NO is necessary for full germination, and it is known to disrupt dormancy (Zhang and Fernie et al. 2023). The application of NO donors to dormant barley seeds induces germination, whereas the application of NO scavengers reinforces and induces dormancy (Albertos et al. 2015). However, barley plants that overexpress *Pgb1* can germinate under hypoxia (Cochrane et al. 2017) and show a higher ATP to ADP ratio, suggesting a prevalent role of the PGB/NO cycle in the energy state.

## Involvement of the N-degron pathway in seed germination

The capacity of Arabidopsis and barley seeds to germinate likely involves N-degron–mediated O_2-_ and NO-sensing mechanisms. This pathway controls the stability of Met-Cys N-terminal proteins, such as ERF-VII, which are degraded by proteasomes when O_2_ and NO are available but are stabilized under O_2_ shortage (Gibbs et al. 2011; Licausi et al. 2011; Weits et al. 2014; White et al. 2017). Some studies have suggested that ERF-VIIs mediate seed germination in Arabidopsis. In fact, the ABI5 transcription factor, which is considered a key repressor of germination, has been found to be a target of Arabidopsis ERF-VII RAP2.3 (Gibbs et al. 2014). Consequently, when O_2_ and NO are unavailable and ERF-VIIs are stabilized in seed, dormancy may be promoted through *ABI5* expression (Signorelli and Considine 2018). In barley, *PRT6* RNAi lines, where N-degron target ERFs are likely stabilized, have been shown a germination impairment (Mendiondo et al. 2016). This finding suggested a similar mechanism to that in *Arabidopsis*. Following the model proposed by (Signorelli and Considine 2018) in the presence of O_2_ and NO during seed imbibition, N-degron targets are degraded; thus, *ABI5* expression is reduced to promote germination. The availability of NO further contributes to direct ABI5 degradation via other ways, including S-nitrosylation (Albertos et al. 2015).

## Can microbiota support seed germination under submergence?

In the last decade, it has become evident that the microbial community associated with plants has several beneficial effects such as defense against pathogens, increased tolerance to abiotic stress, and a greater nutrient availability (Hardoim et al. 2015; Sessitsch et al. 2019). This benefit includes the seed stage, where germination is the primary phase for a successful plant community through seedling establishment (Bergmann and Leveau 2022). In fact, endophytic microorganisms transmitted from mother plants to progeny and across plant generations may represent the initial source of inoculum for plants (Barret et al. 2015; Abdelfattah et al. 2022). A recent large-scale meta-analysis of the microbiota available in the seeds of 50 different plant species identified the presence of a variable and a stable microbial fraction (Simonin et al. 2022). While the presence of few dominant taxa in the core group may indicate specificity, the large variable group will represent the influence of different geographical origin and plant genotypes. They thus may provide crucial support under extreme environmental conditions, such as hypoxia and anoxia, and may have great potential for agricultural applications (Eid et al. 2021). Microorganisms colonize the inner and outer seed coats and embryonic tissues (Rodríguez et al. 2018). During late seed maturation, desiccation may favor microorganisms that can tolerate drought stress and produce endospores (Truyens et al. 2015). The intrinsic characteristics of microbes, such as short generation and rapid mutation (Grandaubert et al. 2019), support rapid adaptation to changing environments, such as flooding events (Martínez-Arias et al. 2022). Recent results on the rhizosphere and root microbiota of adult plants under flooding conditions revealed a dramatic shift in the microbial composition toward anaerobic and, in some cases, detrimental groups in parallel with a reduction in beneficial bacteria (Ferrando and Fernández Scavino 2015; Chialva et al. 2020; Francioli et al. 2021, 2022). Investigations on the microbiota composition of rice seeds (Ahumada et al. 2022) have shown that putative endophytic bacteria have the potential to improve germination. In fact, they can hydrolyze starch and produce indole-related compounds in vitro, which may contribute to coleoptile elongation (Ahumada et al. 2022). A recent study analyzed variations in the seed bacterial community in distinct barley accessions after brief flooding (Gómez-Álvarez et al. 2024). The analysis revealed that successfully germinating accessions showed bacterial enrichment in taxa associated with beneficial functions, such as sugar-related pathways. Tolerance to flooding observed in some plant species and/or varieties may thus be in part supported by the microbiota community that harbors favorable activities despite stress. The identification of a beneficial community, especially at the seed germination stage, may help to support flooding tolerance.

## Future perspectives

Plant embryos are surrounded by specific layers that protect them from environmental stressors. This restricts gas exchange and inevitably leads to hypoxia when metabolic activity within the capsule is high (Radchuk and Borisjuk 2014). It is questionable whether the onset of hypoxia during regular seed development and germination should be considered detrimental or a stressor. The fact that mitotically active cell niches tend to be hosted at low-oxygen tension (Ito and Suda 2014; Herzog et al. 2023) supports the view that maintaining the redox environment under tight control is crucial (which a range of reactive oxygen species (ROS) scavenging enzymes alone cannot do). Hypoxia appears to be a well-managed physiological state in both developing and germinating seeds and could even be beneficial for specific traits.

Advances in non-invasive technologies that facilitate the study of hypoxia in medicine (Mirabello et al. 2018) are being adopted to help advance plant science. The hypoxic adaptations at the molecular and biochemical levels need better clarification, as this may be the key to understanding the embryo's ability to cope with real stressors. The latter are caused by environmental events, such as waterlogging, which clearly restricts gas exchange in all the submerged parts of the plant. The growth and yield of most species are constrained by waterlogging. We suggest that more research is needed on investigating wetland species that are well adapted to growing underwater. Although their strategies have been the focus of previous research, they still hold many potential keys to how plants can cope with too little oxygen.

**OUTSTANDING QUESTIONS BOX**

- Are there specific Met-Cys proteins that respond to hypoxia in the context of seed development and germination?
- Is N-degron involved in the mechanism of starch degradation in cereal seeds under hypoxia?
- Is the energy mediator TOR involved in mediating the response to hypoxia in the context of seed development and germination?
- Why do maize plants use natural endogenous hypoxia to slow seed development?
- Void networks represent (functionally) a type of aerenchyma for seeds/fruits. Can we modify these structural components with benefits for seed/fruit quality?
- Hypoxia is intimately linked to energy metabolism. Which cellular processes (specific storage functions?) are most constrained by energy flux under hypoxia?
- Does the seed endophytic microbiome affect the occurrence of and/or adjustment to hypoxia during development or germination?
- How is the microbiome shaped by hypoxia during seed development?

## Author contributions

C.P. and H.R. conceived the manuscript. All the authors contributed to the writing of the manuscript. C.P. and H.R. reviewed the final version of the manuscript.

## Data Availability

No new data were generated or analysed in support of this research.

## Dive Curated Terms

The following phenotypic, genotypic, and functional terms are of significance to the work described in this paper:

TIR1 Gramene: Os05g0150500
TIR1 Araport: Os05g0150500

ZPR2 Gramene: AT3G60890
ZPR2 Araport: AT3G60890

AUX1 Gramene: Os01g0856500
AUX1 Araport: Os01g0856500

PRT6 Gramene: At5g02310
PRT6 Araport: At5g02310

MYBS1 Gramene: Os01g0524500
MYBS1 Araport: Os01g0524500

CBL10 Gramene: Os01g0711500
CBL10 Araport: Os01g0711500

AFB2 Gramene: Os04g0395600
AFB2 Araport: Os04g0395600

VRN2 Gramene: At4g16845
VRN2 Araport: At4g16845

ABI5 Gramene: AT2G36270
ABI5 Araport: AT2G36270
